# Carbohydrate Antigen 125 Is a Biomarker of the Severity and Prognosis of Pulmonary Hypertension

**DOI:** 10.3389/fcvm.2021.699904

**Published:** 2021-07-20

**Authors:** Yi Zhang, Qi Jin, Zhihui Zhao, Qing Zhao, Xue Yu, Lu Yan, Xin Li, Anqi Duan, Chenhong An, Xiuping Ma, Changming Xiong, Qin Luo, Zhihong Liu

**Affiliations:** ^1^Center for Pulmonary Vascular Diseases, Fuwai Hospital, National Center for Cardiovascular Diseases, Chinese Academy of Medical Sciences and Peking Union Medical College, Beijing, China; ^2^Department of Cardiology, Shanghai Institute of Cardiovascular Diseases, Zhongshan Hospital, Fudan University, Shanghai, China; ^3^Department of Cardiology, Qingdao Municipal Hospital, Qingdao, China

**Keywords:** pulmonary hypertension, carbohydrate antigen 125, biomarkers, prognosis, severity

## Abstract

**Background:** Emerging evidence has showed that serum carbohydrate antigen 125 (CA 125) levels are associated with the severity and prognosis of heart failure. However, its role in pulmonary hypertension remains unclear. This study aimed to investigate the clinical, echocardiographic, hemodynamic, and prognostic associations of CA 125 in pulmonary hypertension.

**Methods and Results:** We conducted a retrospective cohort study of all idiopathic pulmonary arterial hypertension and chronic thromboembolic pulmonary hypertension patients receiving CA 125 measurement in Fuwai Hospital (January 1, 2014–December 31, 2018). The primary end-point was cumulative 1-year clinical worsening-free survival rate. Linear regression was performed to assess the association between CA 125 and clinical, echocardiographic, and hemodynamic parameters. Cox proportional hazards models were used to assess the association between CA 125 and clinical worsening events. Receiver operating characteristic (ROC) curve analysis was performed to determine the predictive performance of CA 125. A total of 231 patients were included. After adjustment, CA 125 still positively correlated with World Health Organization functional class, NT-proBNP, right ventricular end-diastolic diameter, pericardial effusion, mean right atrial pressure and pulmonary arterial wedge pressure; negatively correlated with 6-min walk distance, left ventricular end-diastolic diameter, mixed venous oxygen saturation, and cardiac index. After adjustment, CA 125 > 35 U/ml was associated with over 2 folds increased risk of 1-year clinical worsening. Further, ROC analysis showed that CA 125 provided additional predictive value in addition to the established pulmonary hypertension biomarker NT-proBNP.

**Conclusion:** CA 125 was associated with functional status, echocardiography, hemodynamics and prognosis of pulmonary hypertension.

## Introduction

Carbohydrate antigen 125 (CA 125), also known as mucin 16, is a glycoprotein synthesized by serosal cells in response to mechanical stress (congestion) or inflammatory stimuli (1–3). High serum CA 125 levels have been identified in malignancies such as ovarian, lung and gastrointestinal cancer (4). Currently, CA 125 is a widely used biomarker for the screening (5), monitoring (6) and risk stratification (7) of ovarian cancer. In addition, emerging evidence has linked serum CA 125 levels to non-malignant conditions such as cardiovascular disease (e.g., heart failure, pericardial diseases, and coronary artery disease) (8). More specifically, serum CA 125 levels were found to be associated with functional class (9), echocardiography (10), and hemodynamics (11) in heart failure. Furthermore, some studies have demonstrated the diagnostic and prognostic value of CA 125 in heart failure (12, 13). The capability of CA 125 to serve as a therapeutic target for heart failure has also been investigated, and the results were promising (14, 15).

As the release of CA 125 is irrelevant to the etiology of cardiac aggression, it should be considered a final organ damage marker (8). Thus, it may also play a role in pulmonary hypertension (PH). Unfortunately, there is still limited knowledge on this topic. Rahimi-Rad et al. reported that patients with PH had higher serum CA 125 levels than those without PH in chronic obstructive pulmonary disease (16). A similar phenomenon was also observed in congenital heart disease (17). Whether serum CA 125 levels are correlated with the severity and prognosis of PH remains unclear. In the present study, we aimed to investigate the correlations between CA125 and the functional status, echocardiography, hemodynamics, and prognosis of PH in a retrospective cohort.

## Materials and Methods

### Study Design and Participants

This observational retrospective cohort study was conducted at Fuwai Hospital, Chinese Academy of Medical Sciences (Beijing, China). We screened all patients with idiopathic pulmonary arterial hypertension (IPAH) and chronic thromboembolic pulmonary hypertension (CTEPH) who underwent right heart catheterization (RHC) from January 1, 2014, to December 31, 2018. Patients with CA 125 data and multiple clinical visit/hospitalization records were enrolled as long as they had a minimum of 1 year of follow-up data for outcomes. In addition, echocardiography-suspected PH patients with normal invasive pulmonary arterial pressure and CA 125 data were also recruited as the control group. The establishment of IPAH and CTEPH was based on the 2009 (before January 2016) or 2015 European Society of Cardiology/European Respiratory Society (ERS) guidelines (18, 19). Normal pulmonary arterial pressure was defined as the mean pulmonary arterial pressure (mPAP) <25 mm Hg (18, 19). By design, patients were excluded if they had (1) any malignancy, (2) inflammatory diseases, or (3) active infection. The following clinical data were collected via an electronic medical record system by two independent reviewers: demographics, etiology of PH, 6-minute walk distance (6MWD), N-terminal pro-brain natriuretic peptide (NT-proBNP) levels, smoking history, alcohol consumption, World Health Organization functional class (WHO-FC), PH-specific medication, history of balloon pulmonary angioplasty/pulmonary endarterectomy, comorbidities, parameters derived from echocardiography and RHC, serum CA 125 levels, and follow-up data. The study protocol was approved by the Ethics Committee of Fuwai Hospital. Written informed consent was obtained from each patient.

### CA 125 Measurement

Fasting venous blood samples were collected for CA 125 measurement on the first day of admission. Serum levels of CA125 were measured using a chemiluminescent microparticle immunoassay (product name: Access OV Monitor; Cat. No. 386357; Beckman Coulter Inc., Brea, CA, USA). Please refer to the manufacturer's website for the detailed methodology of the Access OV Monitor (https://mms.mckesson.com/product/586335/Beckman-Coulter-386357). The upper limit of normal for CA 125 was 35 U/ml with the Access OV Monitor. Accordingly, the included patients were divided into either the CA 125 > 35 U/ml group or the CA 125 ≤ 35 U/ml group.

### RHC and Echocardiographic Examination

The detailed protocol for RHC has been provided in our previous publications (20–23). Briefly, with local anesthesia under continuous electrocardiographic monitoring, a 6 French pigtail catheter or 7 French Swan-Ganz catheter (Edwards Lifesciences World Trade Co., Ltd, Irvine, CA, USA) was advanced into the pulmonary artery through the right femoral vein or right internal jugular vein by placement of a 6 or 7 French vascular sheath. Correct catheter positioning was verified by fluoroscopy. Transducers were positioned at the midaxillary line and zeroed at atmospheric pressure. Transthoracic echocardiography was performed by experienced ultrasonologists in the Department of Echocardiography under the current guidelines (24).

### Outcome

We considered the cumulative 1-year clinical worsening-free survival rate as the primary endpoint. Clinical worsening was defined as the occurrence of any of the following events: deteriorated WHO-FC, escalation of PH-specific therapy and rehospitalization due to heart failure or progression of PH. End-point events were adjudicated by two senior clinicians. Any discordance was resolved by the supervisors (QL and ZHL).

### Statistical Analysis

Continuous variables are presented as the mean ± standard deviation. Categorical variables are given as counts. Comparisons between two groups were performed using an independent-sample *t*-test, the Mann–Whitney *U*-test or the chi-square test, as appropriate. Correlations between CA 125 and other variables were examined using the Spearman correlation coefficient. To adjust for potential confounding factors, associations with *P* < 0.100 were further assessed using multivariate linear regression analysis (enter method).

The Kaplan–Meier method was used to assess differences in the rate of 1-year clinical worsening events between patients with values above or below 35 U/ml; curves were compared with the log-rank test. The association between serum CA 125 levels and clinical worsening events was evaluated by a Cox proportional hazards model. Univariate Cox analysis was first performed to screen all prognostic factors. Variables with clinical significance or *P* < 0.100 in univariate analysis were selected for multivariate Cox analysis (enter method). We tested the Cox proportional hazards assumption for each covariate using Schoenfeld residuals. The linearity assumption for CA 125 was evaluated by restricted cubic splines with four knots. Collinearity diagnostics were examined for the potential presence of collinearity between independent variables in multivariate linear regression analysis and multivariate Cox analysis. Receiver operating characteristic (ROC) curve analysis was performed to assess the predictive performance of CA 125. Internal validation was performed using 500 bootstrap resamples (25, 26).

Values of CA 125 and NT-proBNP were logarithmically transformed (ln) and then used in correlation analysis, linear regression and the Cox proportional hazards model. No single missing value was replaced. A two-sided *P* < 0.05 was considered indicative of statistical significance. Data analysis was performed using SPSS (version 23.0), R-studio (version 1.4.1106), R (version 4.0.5), and MedCalc (version 19.7.2).

## Results

### Patient Enrolment

**Figure 1:**
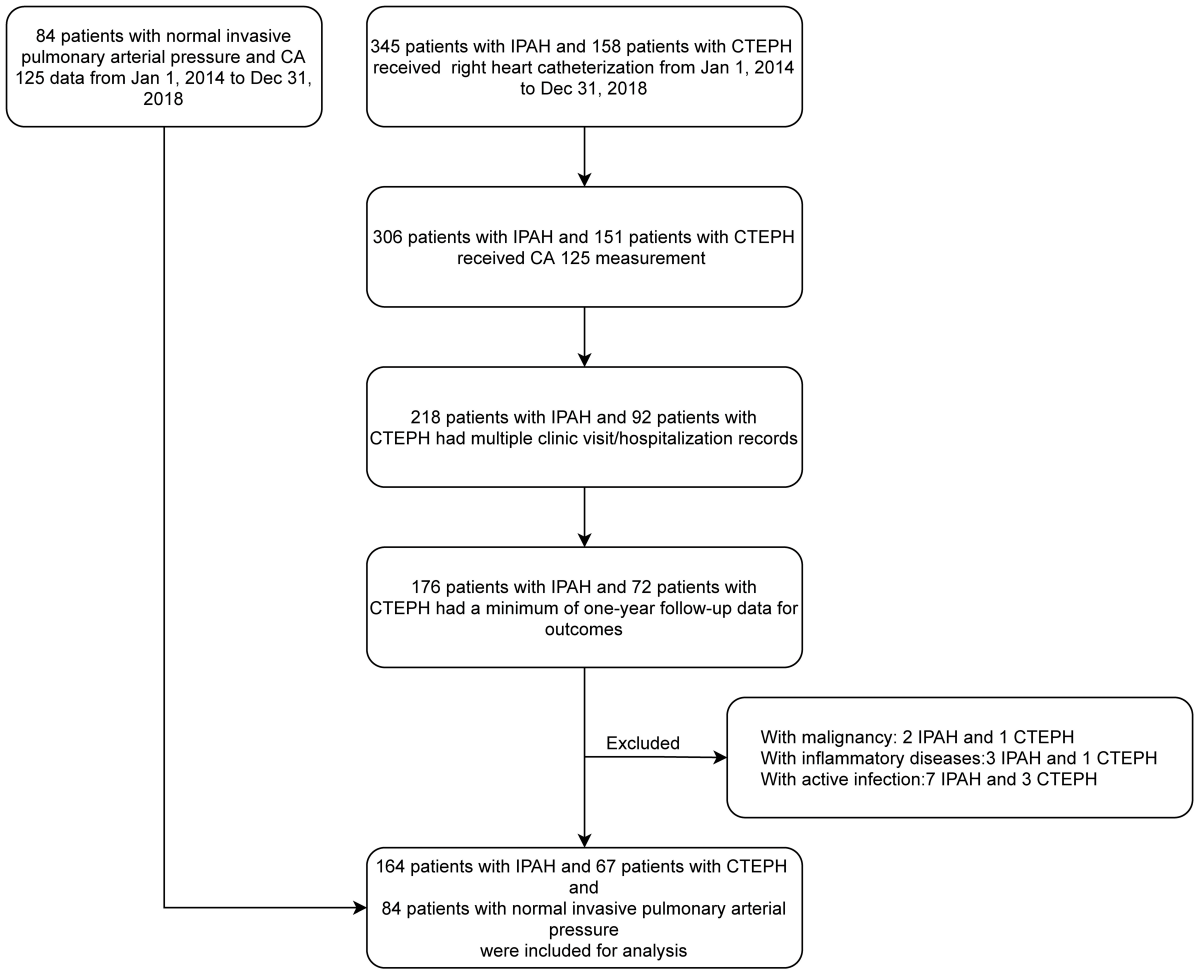
Flow diagram of patient enrollment and exclusion. CA 125, Carbohydrate antigen 125; CTEPH, Chronic thromboembolic pulmonary hypertension; IPAH, Idiopathic pulmonary arterial hypertension.

We identified 231 (45.9%) eligible records for IPAH/CTEPH patients from the 503 records assessed; of the patients, 164 were IPAH and 67 were CTEPH. Furthermore, 84 patients with normal invasive pulmonary arterial pressure and CA 125 data were included as controls. A flow chart displaying the enrolment process is shown in Figure 1.

### Baseline Characteristics

The baseline characteristics of all included patients are presented in Table 1. Among 231 patients with PH, 191 were categorized into the CA 125 ≤ 35 U/ml group and 40 into the CA 125 > 35 U/ml group. At baseline, 111 (48.1%) of 231 patients presented with WHO-FC III/IV, and 40 (17.3%) patients did not receive PH-specific medication. During the follow-up period, 73 (31.6%) patients experienced clinical worsening events. More specifically, 20 patients had deteriorated WHO-FC, 16 patients escalated their PH-specific therapy, and 37 patients were rehospitalized due to heart failure or progression of pulmonary hypertension. Among patients who experienced clinical worsening, 23 were in the CA 125>35 U/ml group, and 50 were in the CA 125 ≤ 35 U/ml group.

**Table 1:** Basic characteristics of control group and patients with PH.

**Variables**	**Control (*n* = 84)**	**IPAH/CTEPH**			*P*-value^*^
		**Total (*n* = 231)**	**CA 125 ≤ 35 U/ml (*n* = 191)**	**CA 125 > 35 U/ml (*n* = 40)**	
Age, years	50.4 ± 16.2	40.0 ± 15.30^‡^	40.2 ± 15.2^‡^	38.9 ± 16.0^‡^	0.500
Female gender, no.	62 (73.8%)	158 (68.4%)	131 (68.6%)	27 (67.5%)	0.893
BMI, kg/m^2^	23.0 ± 3.9	23.2 ± 3.5	23.3 ± 3.5	22.8 ± 3.4	0.354
IPAH/CTEPH, no.	–	164/67	136/55	28/12	0.879
6 MWD, m	454.4 ± 96.2	420.4 ± 100.4^†^	424.0 ± 101.0	398.3 ± 95.2^‡^	0.259
NT-proBNP, pg/ml	135.0 (53.3, 314.9)	880.0 (170.3, 1908.0)^‡^	712.9 (151.5, 1705.0)^‡^	1,375.0 (888.7, 3206.5)^‡^	**0.002**
Smoking, no.	12 (14.3%)	29 (12.6%)	21 (11.0%)	8 (20.0%)	0.118
Alcohol intake, no.	13 (15.5%)	22 (9.5%)	16 (8.4%)	6 (15.0%)	0.194
WHO-FC		^‡^	^‡^	^‡^	**0.007**
I or II, no.	71 (84.5%)	120 (51.9%)	107 (56.0%)	13 (32.5%)	
III or IV, no.	13 (15.5%)	111 (48.1%)	84 (44.0%)	27 (67.5%)	
PH specific medication					0.376
None, no.	–	40 (17.3%)	35 (18.3%)	5 (12.5%)	
Mono or combination therapy, no.	–	191 (82.7%)	156 (81.7%)	35 (87.5%)	
PEA or BPA^#^, no.	–	24 (10.4%)	23 (12.0%)	1 (2.5%)	**0.044**
**Co-morbidities**					
Systemic hypertension, no.	25 (29.8%)	45 (19.5%)	40 (20.9%)	5 (12.5%)^†^	0.220
Diabetes mellitus, no.	4 (4.8%)	11 (4.8%)	9 (4.7%)	2 (5.0%)	1.000
Hyperlipidemia, no.	15 (17.9%)	22 (9.5%)^†^	22 (11.5%)	0^†^	**0.017**
**Echocardiography**					
LVEF, %	63.4 ± 6.4	63.3 ± 5.8	63.3 ± 5.8	63.0 ± 5.7	0.785
LA, mm	34.3 ± 5.9	31.0 ± 5.5^‡^	30.8 ± 5.0^‡^	31.8 ± 7.5^‡^	0.722
LVED, mm	44.5 ± 4.7	37.8 ± 6.3^‡^	38.2 ± 6.2^‡^	36.2 ± 6.6^‡^	**0.064**
RVED, mm	25.6 ± 6.6	32.2 ± 6.6^‡^	31.2 ± 6.4^‡^	33.7 ± 6.5^‡^	** <0.001**
sPAP, mm Hg	47.0 ± 10.0	86.7 ± 26.0^‡^	87.85 ± 27.2^‡^	81.1 ± 18.9^‡^	**0.096**
Pericardial effusion, no.	7 (8.3%)	32 (13.9%)	18 (9.4%)	14 (35.0%)^‡^	** <0.001**
**Hemodynamics**					
S_V_O_2_, %	76.0 ± 5.8	67.1 ± 6.5^‡^	67.8 ± 6.2^‡^	63.9 ± 7.4^‡^	** <0.001**
mRAP, mm Hg	3.0 (1.0, 5.0)	5.0 (2.0, 8.0)^‡^	4.0 (2.0, 7.0)^‡^	7.0 (3.3, 13.8)^‡^	**0.001**
mPAP, mm Hg	15.2 ± 3.2	51.2 ± 13.4^‡^	51.3 ± 13.5^‡^	50.9 ± 13.3^‡^	0.791
CI, L/min/m^2^	3.7 ± 0.8	3.0 ± 1.0^‡^	3.1 ± 1.0^‡^	2.7 ± 0.9^‡^	**0.018**
PVR, Wood units	1.2 ± 0.8	10.7 ± 5.1^‡^	10.6 ± 5.2^‡^	11.3 ± 4.4^‡^	0.227
PAWP, mm Hg	8.3 ± 3.3	7.7 ± 3.4	7.6 ± 3.2	8.5 ± 4.2	0.138
CA125, U/ml	9.5 (6.1, 18.2)	17.3 (11.3, 25.8)^‡^	14.5 (10.6, 21.1)^‡^	55.9 (43.7, 83.0)^‡^	** <0.001**

*Data are presented as mean ± standard deviation, median (range) or number (percentage). BMI, Body mass index; BPA, balloon pulmonary angioplasty; CA 125, Carbohydrate antigen 125; CI, Cardiac index; CTEPH, Chronic thromboembolic pulmonary hypertension; IPAH, Idiopathic pulmonary arterial hypertension; LA, Left atrium dimension; LVED, Left ventricular end-diastolic diameter; LVEF, Left ventricular ejection fraction; mPAP, Mean pulmonary arterial pressure; mRAP, Mean right atrial pressure; NT-proBNP, N-terminal pro-brain natriuretic peptide; PAWP, Pulmonary arterial wedge pressure; PAH, Pulmonary arterial hypertension; PEA, Pulmonary endarterectomy; PH, Pulmonary hypertension; PVR, Pulmonary vascular resistance; RVED, Right ventricular end-diastolic diameter; 6MWD, 6-min walk distance; sPAP, Systolic pulmonary arterial pressure; S_V_O_2_, Mixed venous oxygen saturation; WHO-FC, World Health Organization functional class*.

# *Only for patients with CTEPH*.

* *CA 125 > 35 U/ml compared with CA 125 ≤ 35 U/ml*.

† *P < 0.05, compared with control group*.

‡ *P < 0.001, compared with control group. Bold values means their P value < 0.100*.

### Patients With PH vs. Control Group

Compared to those in the control group, patients with PH were younger (40.0 ± 15.3 vs. 50.4 ± 16.2 years, *P* < 0.001), had worse WHO-FC, 6MWD, echocardiographic and haemodynamic parameters, and had higher serum levels of NT-proBNP and CA 125 [17.3 (11.3, 25.8) vs. 9.5 (6.1, 18.2) U/ml, *P* < 0.001].

### PH Patients With CA 125 > 35 U/ml vs. Those With CA 125 ≤ 35 U/ml

Compared to those with CA 125 ≤ 35 U/ml, patients with CA 125 > 35 U/ml had lower mixed venous oxygen saturation (S_V_O_2_), lower cardiac index (CI) values, a larger right ventricular end-diastolic diameter (RVED), higher prevalence rates of hyperlipidaemia and pericardial effusion, higher mean right atrial pressure (mRAP), and higher serum levels of NT-proBNP and CA 125 [55.9 (43.7, 83.0) vs. 14.5 (10.6, 21.1) U/ml, *P* < 0.001]. In addition, patients with CA 125 > 35 U/ml tended to have a smaller left ventricular end-diastolic diameter (LVED) (36.2 ± 6.6 vs. 38.2 ± 6.2 mm, *P* = 0.064).

### CA 125 Is Weakly Associated With Established Markers of PH Severity

As shown in Table 2, ln(CA 125) was weakly correlated with 6MWD, WHO-FC, ln(NT-proBNP), echocardiographic parameters (LVED, RVED, and pericardial effusion), and haemodynamic parameters [S_v_O_2_, mRAP, CI and pulmonary vascular resistance (PVR)]. In addition, ln(CA 125) tended to correlate with pulmonary arterial wedge pressure (PAWP) (*r* = 0.123, *P* = 0.062). However, no correlations were observed between ln(CA 125) and left atrial dimension (*r* = −0.066, *P* = 0.318), left ventricular ejection fraction (*r* = 0.034, *P* = 0.605), systolic pulmonary arterial pressure (*r* = −0.037, *P* = 0.585), or mPAP (*r* = 0.106, *P* = 0.109). Similar results were observed in the CTEPH and IPAH subgroups (Supplementary Tables 1, 2).

**Table 2:** Correlations between carbohydrate antigen 125 and established markers of PH severity.

**Variables**	**Coefficient (r)**	***P*-value**	Adjusted coefficient (r)^*^	***P*-value**
6MWD	−0.168	**0.018**	−0.208	**0.004**
WHO-FC	0.277	** <0.001**	0.293	** <0.001**
ln (NT-proBNP)	0.309	** <0.001**	0.284	** <0.001**
**Echocardiography**				
LVEF	0.034	0.605		
LA	−0.066	0.318		
LVED	0.215	**0.001**	−0.173	**0.017**
RVED	0.306	** <0.001**	0.382	** <0.001**
sPAP	−0.037	0.585		
Pericardial effusion	0.251	** <0.001**	0.290	** <0.001**
**Hemodynamics**				
S_v_O_2_	−0.230	** <0.001**	−0.312	** <0.001**
mRAP	0.244	**0.001**	0.372	** <0.001**
mPAP	0.106	0.109		
Cardiac index	−0.243	** <0.001**	−0.208	**0.002**
PVR	0.198	**0.003**	0.127	**0.068**
PAWP	0.123	**0.062**	0.157	**0.018**

*ln, Logarithmically transformed; LA, Left atrium dimension; LVED, Left ventricular end-diastolic diameter; LVEF, Left ventricular ejection fraction; mRAP, Mean right atrial pressure; NT-proBNP, N-terminal pro-brain natriuretic peptide; PAWP, Pulmonary arterial wedge pressure; PH, Pulmonary hypertension; PVR, Pulmonary vascular resistance; RVED, Right ventricular end-diastolic diameter; 6MWD, 6-min walk distance; S_V_O_2_, Mixed venous oxygen saturation; WHO-FC, World Health Organization functional class*.

* *Each variable is adjusted for age, gender, body mass index by multivariate linear regression analysis. Bold values means their P value < 0.100*.

In multivariate linear regression analysis (enter method), we further assessed correlations between CA 125 and functional status (6MWD, WHO-FC, and NT-proBNP) and echocardiographic (LVED, RVED and pericardial effusion) and haemodynamic (S_v_O_2_, mRAP, CI, PVR, and PAWP) parameters by adjusting for age, sex, and body mass index. The results showed that ln(CA 125) was still positively correlated with WHO-FC, ln(NT-proBNP), RVED, pericardial effusion, mRAP, and PAWP and negatively correlated with 6MWD, LVED, S_v_O_2_, and CI (Table 2). Similar results were observed in the CTEPH and IPAH subgroups (Supplementary Tables 1, 2). No problems with collinearity were detected in multivariate linear regression analysis (variance inflation factor <5).

### CA 125 Is Associated With Prognosis of PH

**Figure 2:**
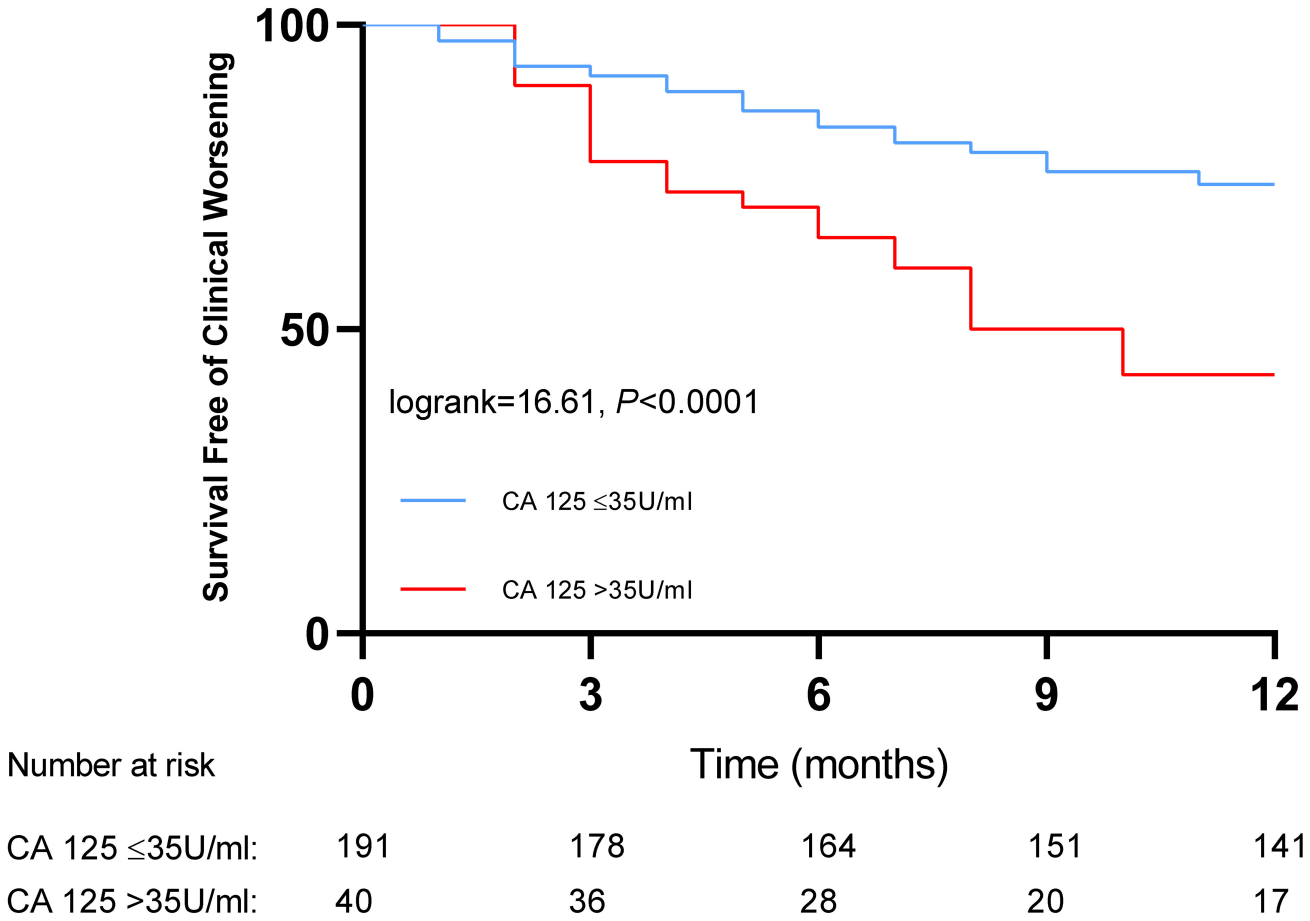
Kaplan-Meier curves for patients with pulmonary hypertension classified by baseline serum levels of carbohydrate antigen 125.

Kaplan–Meier analysis showed that IPAH/CTEPH patients with CA 125 > 35 U/ml had a lower cumulative one-year clinical worsening-free survival rate than those with CA 125 ≤ 35 U/ml (42.5 vs. 73.8%, *P* < 0.0001; Figure 2).

In univariate Cox analysis, 6MWD, ln(NT-proBNP), S_V_O_2_, mRAP, PAWP, and CA 125 > 35 U/ml had a *P* < 0.100 (Table 3). Considering their clinical importance, age, WHO-FC and pericardial effusion were also selected for multivariate Cox analysis (enter method). Events per variable are often used to estimate the sample size needed in multiple Cox analyses, and the lowest acceptable number of events per variable is usually considered to be 10 (27). Given that 73 patients reached the primary endpoint in the present study, it was relatively safe for us to include a maximum of 7 independent variables into multivariate Cox analysis. Model 1 was adjusted for S_v_O_2_, 6MWD, ln(NT-proBNP), mRAP, and PAWP. Model 2 was adjusted for the variables in model 1 plus age. Model 3 was adjusted for the variables in model 1 plus hyperlipidemia. Model 4 was adjusted for the variables in model 1 plus WHO-FC. Model 5 was adjusted for the variables in model 1 plus pericardial effusion. In all 5 Cox models, CA 125 was found to be an independent predictor of clinical worsening (Table 4). The C statistic was 0.648 [95% CI: 0.577–0.718] for model 1, 0.652 [95% CI: 0.582–0.722] for model 2, 0.649 [95% CI: 0.578–0.720] for model 3, 0.649 [95% CI: 0.579–0.720] for model 4 and 0.647 [95% CI: 0.576–0.718] for model 5. Using bootstrap validation, the optimism-corrected C statistic was 0.609 for model 1, 0.606 for model 2, 0.603 for model 3, 0.604 for model 4 and 0.602 for model 5, indicating that the predictive ability of the models is relatively stable in future patients. We did not observe statistically significant deviations from the proportional hazards assumption in any of the Cox models (Supplementary Table 3). When modeled as restricted cubic splines, CA 125 showed a linear association with the HR for clinical worsening (Supplementary Figure 1). No problems with collinearity were detected in multivariate Cox analysis (variance inflation factor <5). Subgroup analysis also showed that CA 125 was an independent predictor of clinical worsening in CTEPH and IPAH (Supplementary Tables 4–7).

**Table 3:** Univariate cox analysis of proportional risks for 1-year clinical worsening.

**Variable**	**β**	**Standard error**	**HR (95% CI)**	**Wald**	***P-*value**
Age	0.009	0.007	1.009 (0.994–1.024)	1.470	0.225
Female gender	−0.094	0.249	0.910 (0.559–1.484)	0.811	0.707
6MWD	−0.002	0.001	0.998 (0.995–1.000)	3.840	**0.050**
ln (NT-proBNP)	0.146	0.085	1.157 (0.979–1.368)	2.918	**0.088**
WHO-FC	0.177	0.234	1.193 (0.754–1.888)	0.568	0.451
Smoking	0.289	0.327	1.335 (0.703–2.535)	0.778	0.378
Alcohol intake	0.048	0.398	1.105 (0.482–2.288)	0.015	0.903
Systemic hypertension	−0.123	0.306	0.884 (0.485–1.611)	0.162	0.687
Diabetes mellitus	−0.662	0.717	0.516 (0.127–2.104)	0.852	0.356
Hyperlipidemia	−0.380	0.463	0.684 (0.276–1.695)	0.674	0.412
LVEF	0.000	0.020	0.982 (0.961–1.041)	0.001	0.982
LA	0.004	0.021	1.004 (0.963–1.047)	0.034	0.854
LVED	−0.016	0.019	0.985 (0.949–1.021)	0.695	0.404
RVED	0.016	0.017	1.017 (0.983–1.051)	0.949	0.330
sPAP	0.005	0.004	1.005 (0.998–1.013)	1.829	0.176
Pericardial effusion	0.410	0.306	1.507 (0.827–2.746)	1.797	0.180
S_V_O_2_	−0.061	0.018	0.941 (0.909–0.974)	12.023	**0.001**
mRAP	0.090	0.023	1.095 (1.047–1.145)	15.558	** <0.001**
mPAP	0.004	0.008	1.004 (0.988–1.020)	0.218	0.641
CI	−0.145	0.135	0.865 (0.664–1.127)	1.156	0.282
PVR	0.016	0.022	1.016 (0.974–1.061)	0.568	0.451
PAWP	0.064	0.035	1.067 (0.996–1.142)	3.411	**0.065**
CA 125 (category^#^)	0.975	0.253	2.650 (1.615–4.349)	14.866	** <0.001**

*CA 125, Carbohydrate antigen 125; CI, Cardiac index; HR, Hazard ratio; LA, Left atrium dimension; LVED, Left ventricular end-diastolic diameter; LVEF, Left ventricular ejection fraction; ln, Logarithmically transformed; mPAP, Mean pulmonary arterial pressure; mRAP, Mean right atrial pressure; NT-proBNP, N-terminal pro-brain natriuretic peptide; PAWP, Pulmonary arterial wedge pressure; PVR, Pulmonary vascular resistance; RVED, Right ventricular end-diastolic diameter; 6MWD, 6-min walk distance; sPAP, Systolic pulmonary arterial pressure; S_V_O_2_, Mixed venous oxygen saturation; WHO-FC, World Health Organization functional class*.

# *CA 125 is classified into two groups, namely CA 125 ≤ 35 U/ml and CA 125 >35 U/ml. Bold values means their P value < 0.100*.

**Table 4:** Multivariate cox analysis of proportional risks for 1-year clinical worsening.

**Model**	**Variable**	**β**	**HR (95% CI)**	***P-*value**
1	CA 125 (category^#^)	0.860	2.362 (1.286–4.340)	**0.006**
	S_v_O_2_	−0.052	0.949 (0.904–0.996)	**0.035**
	6MWD	−0.001	0.999 (0.996–1.002)	0.576
	ln (NT-proBNP)	−0.042	0.959 (0.800–1.150)	0.650
	mRAP	0.009	1.009 (0.942–1.080)	0.802
	PAWP	0.040	1.041 (0.967–1.120)	0.282
2	CA 125 (category^#^)	0.876	2.401 (1.303–4.422)	**0.005**
	S_v_O_2_	−0.049	0.952 (0.906–1.000)	**0.050**
	6MWD	−0.001	0.999 (0.997–1.002)	0.691
	ln (NT-proBNP)	−0.037	0.964 (0.802–1.158)	0.692
	mRAP	0.011	1.011 (0.944–1.083)	0.759
	PAWP	0.037	1.037 (0.965–1.115)	0.323
	Age	0.007	1.007 (0.990–1.024)	0.424
3	CA 125 (category^#^)	1.474	4.366 (1.306–14.590)	**0.017**
	S_v_O_2_	−0.028	0.972 (0.883–1.071)	0.570
	6MWD	−0.003	0.997 (0.991–1.002)	0.225
	ln (NT-proBNP)	−0.153	0.858 (0.603–1.221)	0.396
	mRAP	−0.013	0.987 (0.878–1.110)	0.826
	PAWP	0.003	1.003 (0.883–1.138)	0.969
	Hyperlipidemia	−0.856	0.425 (0.088–2.043)	0.285
4	CA 125 (category^#^)	0.914	2.494 (1.339–4.645)	**0.004**
	S_v_O_2_	−0.054	0.948 (0.902–0.995)	**0.031**
	6MWD	−0.001	0.999 (0.996–1.002)	0.526
	ln (NT-proBNP)	−0.020	0.980 (0.810–1.186)	0.835
	mRAP	0.012	1.012 (0.944–1.084)	0.741
	PAWP	0.037	1.038 (0.963–1.118)	0.331
	WHO-FC	−0.244	0.784 (0.441–1.393)	0.406
5	CA 125 (category^#^)	0.843	2.323 (1.245–4.333)	**0.008**
	S_v_O_2_	−0.053	0.949 (0.904–0.996)	**0.034**
	6MWD	−0.001	0.999 (0.996–1.002)	0.607
	ln (NT-proBNP)	−0.048	0.953 (0.789–1.151)	0.616
	mRAP	0.010	1.010 (0.943–1.081)	0.783
	PAWP	0.040	1.040 (0.967–1.120)	0.289
	Pericardial effusion	0.095	1.100 (0.516–2.342)	0.806

*CA 125, Carbohydrate antigen 125; HR, Hazard ratio; ln, logarithmically transformed; mRAP, Mean right atrial pressure; NT-proBNP, N-terminal pro-brain natriuretic peptide; PAWP, Pulmonary arterial wedge pressure; 6MWD, 6-min walk distance; S_V_O_2_, Mixed venous oxygen saturation; WHO-FC, World Health Organization functional class*.

# *CA 125 is classified into two groups, namely CA 125 ≤ 35 U/ml and CA 125 >35 U/ml. Bold values means their P value < 0.100*.

### CA 125 Provided Additional Predictive Value in Combination With NT-proBNP

**Figure 3:**
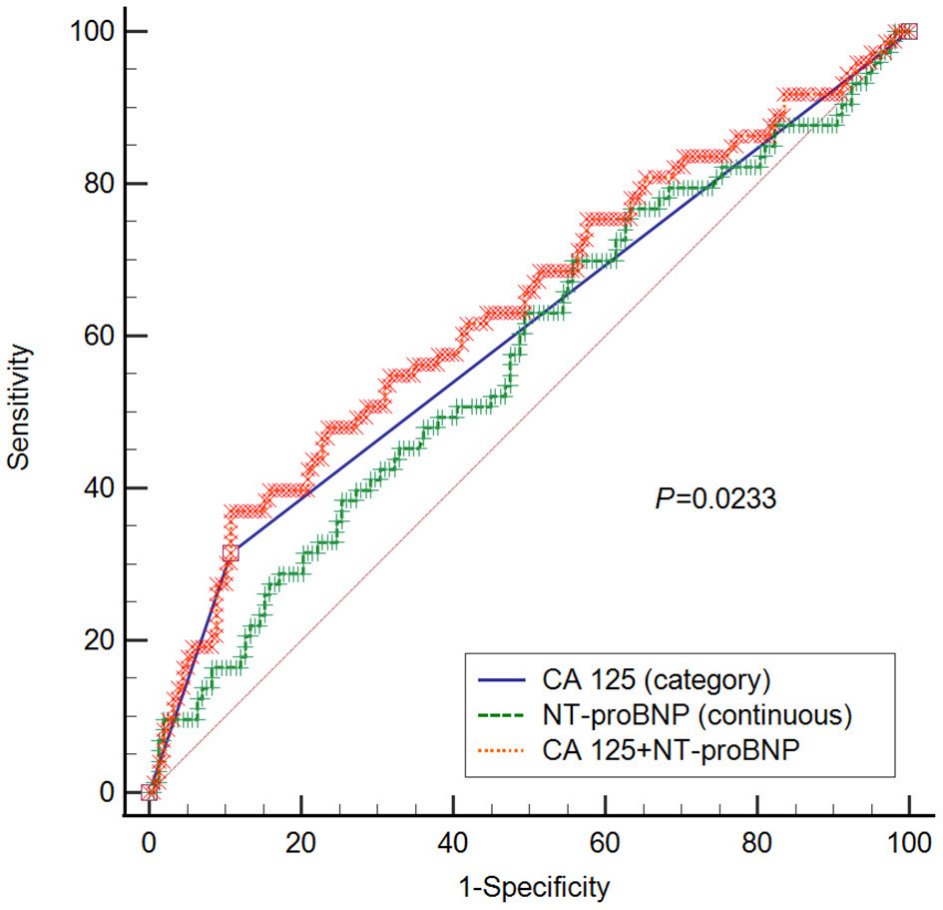
ROC curve for CA 125, NT-proBNP, and CA 125+ NT-proBNP in predicting clinical worsening. *P*-value refers to the comparison between NT-proBNP and NT-proBNP + CA 125. No statistical significances were observed between CA 125 and NT-proBNP (*P* = 0.5108), or CA 125 and CA 125 + NT-proBNP (*P* = 0.2710). CA 125 is classified into two groups, namely CA 125 ≤ 35 U/ml and CA 125 > 35 U/ml; CA 125, Carbohydrate antigen 125; NT-proBNP, N-terminal pro-brain natriuretic peptide.

To provide better insight into the predictive value of CA 125 for clinical worsening, we benchmarked it against the established PH biomarker NT-proBNP (18). The areas under the curve for CA 125, NT-proBNP, and combined CA 125 and NT-proBNP were 0.604 (95% CI 0.537–0.667), 0.573 (95% CI 0.507–0.638), and 0.637 (0.571–0.699), respectively. The area under the curve of CA 125 + NT-proBNP was significantly higher than that of NT-proBNP alone (*P* = 0.0233), as shown in Figure 3. No significant differences were observed between CA 125 and NT-proBNP (*P* = 0.5108) or CA 125 and CA 125 + NT-proBNP (*P* = 0.2710).

## Discussion

In the present study, we found that serum CA 125 levels were weakly correlated with functional status (6MWD, WHO-FC, and NT-proBNP) and echocardiographic (LVED, RVED, and pericardial effusion) and haemodynamic (S_v_O_2_, mRAP, and CI) parameters of PH after adjustment. Moreover, CA 125>35 U/ml was found to be an independent predictor of 1-year clinical worsening in PH.

### CA 125 Is Weakly Associated With Established Markers of PH Severity

Compared to patients with normal invasive pulmonary arterial pressure, patients with PH had higher serum CA 125 levels, which was consistent with the results seen in chronic obstructive pulmonary disease (16) and congenital heart disease (17).

WHO-FC, 6MWD, NT-proBNP, pericardial effusion, mRAP, CI, and S_v_O_2_ are well-established prognostic markers of IPAH (18). We demonstrated that CA 125 was positively correlated with WHO-FC (9), NT-proBNP (28), RVED (10), pericardial effusion (10), mRAP (9, 11), and PAWP (9, 11) in PH, which was consistent with the results seen in heart failure. Additionally, we also found that CA 125 was negatively correlated with 6MWD, LVED, S_v_O_2_, and CI in PH. Therefore, CA 125 may serve as a novel biomarker of severity in PH.

To date, it remains unclear what leads to CA125 overproduction in heart failure (29, 30). It has been hypothesized to correlate with so-called “stressed” mesothelial cells: (1) mesothelial cells are stimulated by tissue stretching/mechanical stress induced by fluid overload due to heart failure. (2) mesothelial cells are stimulated by inflammatory cytokine network activation (interleukin-1, tumor necrosis factor-α, lipopolysaccharides) (29, 31). PH is characterized by increased mPAP and high PVR, which cause right ventricular hypertrophy, and finally result in right-sided heart failure. Based on our results, we hereby offered a hypothesis to explain the relationship between CA 125 elevation and right heart failure. Elevated PVR increased the afterload of right ventricle, which would further cause right ventricular dilation and elevation of right atrial filling pressure, leading to elevated hydrostatic pressure and congestion, which would further cause both serosal mechanical stretch and third space fluid retention with resultant inflammation and cytokines release (8), ultimately resulting in the elevation of CA 125 (29).

### CA 125 Is Associated With Prognosis of PH

Compared to those with CA 125 ≤ 5 U/ml, patients with CA 125 > 35 U/ml had higher serum levels of NT-proBNP and worse echocardiographic and haemodynamic parameters at baseline. In all 5 Cox models we constructed, CA125 > 35 U/ml was associated with an over 2-fold increased risk of 1-year clinical worsening, which was similar to the results seen in heart failure (32, 33). Furthermore, ROC analysis showed that CA 125 provided additional predictive value in addition to the established PH biomarker NT-proBNP (18). Due to its close relationship with congestion (8), CA 125 should be considered a final organ damage marker in cardiovascular diseases. This may explain why we found that CA 125 was a severe and prognostic marker in PH.

### Clinical Implications

Based on the current knowledge, CA 125 has several merits for use as a biomarker in clinical practice: (1) it is inexpensive, widely available, and measurable with standard methods and has a relatively long half-life (5–7 days) (34–36). (2) It correlates with the severity and prognosis of PH, providing additional information in combination with established risk factors. Therefore, CA 125 may become a valuable tool in the management of PH in the near future. Further studies are needed to evaluate its capacity to monitor response to treatment, serving as a therapeutic target and predicting hard outcomes (such as mortality).

### Limitations

As a retrospective cohort study, follow-up bias is our biggest concern. Two hundred nine patients with CA 125 data were excluded for not having a minimum of 1 year of follow-up data for outcomes (Figure 1). Among these 209 patients, 29 had CA 125 > 35 U/ml, and 180 had CA 125 ≤ 35 U/ml. In other words, these two groups had similar rates of loss to follow-up [29/(40 + 29), 42.0% for CA 125 > 35 U/ml; 180/(191 + 180), 48.5% for CA 125 ≤ 35 U/ml]. Moreover, the percentage of patients who reached the endpoint was higher in the CA 125 > 35 U/ml group (57.5 vs. 26.2%). Taken together, these results indicate that the effect size (e.g., hazard ratio) in the present study would be underestimated rather than exaggerated. Follow-up bias should not undermine our conclusion. The present study included only patients with IPAH/CTEPH, which may limit its generalizability to other etiologies of PH. We planned to conduct a comprehensive retrospective study to investigate the correlations between CA 125 and functional status and echocardiographic and hemodynamic parameters in all five groups of PH patients.

## Conclusion

CA 125 was associated with the functional status, echocardiography and hemodynamics of PH. It was found to be an independent predictor of 1-year clinical worsening in PH. Moreover, it provided additional predictive value in combination with the established PH biomarker NT-proBNP. Given that the number of patients with elevated CA 125 levels was low, our results, despite being promising, need to be confirmed in a large prospective study.

## Data Availability Statement

The original contributions presented in the study are included in the article/Supplementary Material, further inquiries can be directed to the corresponding author/s.

## Ethics Statement

The studies involving human participants were reviewed and approved by the Ethics Committee of Fuwai Hospital. The patients/participants provided their written informed consent to participate in this study.

## Author Contributions

ZL and QL contributed to the conception of the study. YZ and QJ performed the data analyses and wrote the manuscript. ZZ, QZ, and CX contributed significantly to analysis and manuscript preparation. XY, LY, XL, AD, CA, and XM contributed to data collection. All authors critically reviewed the manuscript for intellectual content and had final responsibility for the decision to submit for publication.

## Conflict of Interest

The authors declare that the research was conducted in the absence of any commercial or financial relationships that could be construed as a potential conflict of interest.
