# Supplementary material for: Carbohydrate Antigen 125 Is a Biomarker of the Severity and Prognosis of Pulmonary Hypertension
Source: Front Cardiovasc Med. 2021 Jul 20;8:699904. doi: 10.3389/fcvm.2021.699904 (PMC8330972; doi:10.3389/fcvm.2021.699904)
Supplement: Supplementary file 1 [file Data_Sheet_1.docx]

Figure S1

Title: Cox regression models estimating the predictive value of CA 125 for clinical worsening.

Legends: Model 1 was adjusted for S_v_O_2_, 6MWD, ln (NT-proBNP), mRAP and PAWP. Model 2 was adjusted for the variables in model 1 plus age. Model 3 was adjusted for the variables in model 1 plus hyperlipidemia. Model 4 was adjusted for the variables in model 1 plus WHO-FC. Model 5 was adjusted for the variables in model 1 plus pericardial effusion. CA 125, Carbohydrate antigen 125; HR, Hazard ratio; ln, Logarithmically transformed; mRAP, Mean right atrial pressure; NT-proBNP, N-terminal pro-brain natriuretic peptide; PAWP, Pulmonary arterial wedge pressure; 6MWD, 6-min walk distance; S_V_O_2_, Mixed venous oxygen saturation; WHO-FC, World Health Organization functional class.

Table S1 Correlations between carbohydrate antigen 125 with established markers of CTEPH severity.

| Variables | Coefficient (r) | *P* value | Adjusted coefficient (r) * | *P* value |
| --- | --- | --- | --- | --- |
| 6MWD | -0.341 | **0.010** | -0.343 | **0.019** |
| WHO-FC | 0.364 | **0.002** | 0.344 | **0.004** |
| ln (NT-proBNP) | 0.530 | **<0.001** | 0.284 | **<0.001** |
| Echocardiography |  |  |  |  |
| LVEF | 0.107 | 0.391 |  |  |
| LA | -0.165 | 0.183 |  |  |
| LVED | -0.228 | **0.064** | -0.176 | 0.221 |
| RVED | 0.542 | **<0.001** | 0.441 | **0.001** |
| sPAP | -0.031 | 0.810 |  |  |
| Pericardial effusion | 0.320 | **0.008** | 0.335 | **0.005** |
| Hemodynamics |  |  |  |  |
| S_v_O_2_ | -0.322 | **0.008** | -0.378 | **0.001** |
| mRAP | 0.379 | **0.002** | 0.431 | **<0.001** |
| mPAP | 0.269 | **0.028** | 0.138 | 0.330 |
| Cardiac index | -0.339 | **0.005** | -0.417 | **<0.001** |
| PVR | 0.340 | **0.005** | 0.260 | **0.048** |
| PAWP | 0.346 | **0.004** | 0.313 | **0.010** |

CTEPH, Chronic thromboembolic pulmonary hypertension; ln, logarithmically transformed; LA, Left atrium dimension; LVED, Left ventricular end-diastolic diameter; LVEF, Left ventricular ejection fraction; mPAP, Mean pulmonary arterial pressure; mRAP, Mean right atrial pressure; NT-proBNP, N-terminal pro-brain natriuretic peptide; PAWP, Pulmonary arterial wedge pressure; PVR, Pulmonary vascular resistance; RVED, Right ventricular end-diastolic diameter; 6MWD, 6-min walk distance; sPAP, Systolic pulmonary arterial pressure; S_V_O_2_, Mixed venous oxygen saturation; WHO-FC, World Health Organization functional class. *Each variable is adjusted for age, gender and body mass index by multivariate linear regression analysis.

Table S2 Correlations between carbohydrate antigen 125 with established markers of IPAH severity.

| Variables | Coefficient (r) | *P* value | Adjusted coefficient (r)^#^ | *P* value |
| --- | --- | --- | --- | --- |
| 6MWD | -0.123 | 0.144 |  |  |
| WHO-FC | 0.243 | **0.002** | 0.243 | **0.002** |
| ln (NT-proBNP) | 0.202 | **0.009** | 0.163 | **0.037** |
| Echocardiography |  |  |  |  |
| LVEF | -0.005 | 0.951 |  |  |
| LA | 0.021 | 0.788 |  |  |
| LVED | -0.213 | **0.006** | -0.164 | **0.038** |
| RVED | 0.182 | **0.020** | 0.312 | **<0.001** |
| sPAP | -0.032 | 0.689 |  |  |
| Pericardial effusion | 0.223 | **0.004** | 0.245 | **0.002** |
| Hemodynamics |  |  |  |  |
| S_v_O_2_ | -0.216 | **0.006** | -0.258 | **0.001** |
| mRAP | 0.185 | **0.018** | 0.299 | **<0.001** |
| mPAP | 0.021 | 0.785 |  |  |
| Cardiac index | -0.200 | **0.010** | -0.127 | 0.104 |
| PVR | 0.138 | **0.078** | 0.116 | **0.145** |
| PAWP | 0.031 | 0.697 |  |  |

IPAH, Idiopathic pulmonary arterial hypertension; ln, logarithmically transformed; LA, Left atrium dimension; LVED, Left ventricular end-diastolic diameter; LVEF, Left ventricular ejection fraction; mPAP, Mean pulmonary arterial pressure; mRAP, Mean right atrial pressure; NT-proBNP, N-terminal pro-brain natriuretic peptide; PAWP, Pulmonary arterial wedge pressure; PVR, Pulmonary vascular resistance; RVED, Right ventricular end-diastolic diameter; 6MWD, 6-min walk distance; sPAP, Systolic pulmonary arterial pressure; S_V_O_2_, Mixed venous oxygen saturation; WHO-FC, World Health Organization functional class. ^#^Each variable is adjusted for age, gender, body mass index by multivariate linear regression analysis.

Table S3 Correlation between rank of time and partial residual for independent variables in Cox models.

| Variable | r^#^ | *P* value |
| --- | --- | --- |
| CA 125 (category^$^) | 0.098 | 0.411 |
| S_v_O_2_ | -0.005 | 0.965 |
| 6MWD | -0.067 | 0.602 |
| ln (NT-proBNP) | 0.001 | 0.996 |
| mRAP | 0.033 | 0.783 |
| PAWP | 0.085 | 0.473 |
| Age | 0.062 | 0.602 |
| Hyperlipidemia | -0.115 | 0.334 |
| WHO-FC | 0.061 | 0.608 |
| Pericardial effusion | -0.121 | 0.309 |

CA 125, Carbohydrate antigen 125; ln, Logarithmically transformed; mRAP, Mean right atrial pressure; NT-proBNP, N-terminal pro-brain natriuretic peptide; PAWP, Pulmonary arterial wedge pressure; PH, pulmonary hypertension; 6MWD, 6-min walk distance; S_V_O_2_, Mixed venous oxygen saturation; WHO-FC, World Health Organization functional class. ^#^Pearson correlation coefficient. ^$^ CA 125 is classified into two groups, namely CA 125 ≤35 U/ml and CA 125 >35 U/ml.

Table S4 Univariate cox analysis of proportional risks for one-year clinical worsening in patients with CTEPH.

| Variable | β | standard error | HR (95% CI) | Wald | *P* value |
| --- | --- | --- | --- | --- | --- |
| Age | -0.007 | 0.018 | 0.993 (0.958-1.029) | 0.149 | 0.700 |
| Female gender | -0.320 | 0.408 | 0.726 (0.326-1.616) | 0.615 | 0.433 |
| 6MWD | -0.004 | 0.002 | 0.996 (0.991-1.000) | 3.270 | **0.071** |
| ln (NT-proBNP) | 0.222 | 0.143 | 1.249 (0.944-1.651) | 2.431 | 0.119 |
| WHO-FC | 0.237 | 0.403 | 1.267 (0.575-2.793) | 0.346 | 0.557 |
| PEA or BPA | -0.394 | 0.446 | 0.675(0.282-1.615) | 0.781 | 0.377 |
| Smoking | 0.647 | 0.447 | 1.910 (0.796-4.584) | 2.098 | 0.148 |
| Alcohol intake | -0.018 | 0.616 | 0.983(0.294-3.284) | 0.001 | 0.977 |
| Systemic hypertension | -0.559 | 0.500 | 0.572 (0.214-1.525) | 1.248 | 0.264 |
| Diabetes mellitus | -1.072 | 1.021 | 0.342 (0.046-2.532) | 1.103 | 0.294 |
| Hyperlipidemia | -1.235 | 0.738 | 0.291 (0.069-1.235) | 2.803 | **0.094** |
| LVEF | 0.006 | 0.047 | 1.006 (0.918-1.103) | 0.018 | 0.893 |
| LA | -0.023 | 0.034 | 0.977 (0.914-1.045) | 0.466 | 0.495 |
| LVED | -0.057 | 0.032 | 0.944 (0.886-1.006) | 3.119 | **0.077** |
| RVED | 0.056 | 0.025 | 1.058 (1.007-1.112) | 5.036 | **0.025** |
| sPAP | 0.021 | 0.008 | 1.022 (1.006-1.037) | 7.881 | **0.005** |
| Pericardial effusion | 1.242 | 0.471 | 3.461 (1.375-8.709) | 6.953 | **0.008** |
| S_V_O_2_ | -0.072 | 0.033 | 0.930 (0.872-0.993) | 4.691 | **0.030** |
| mRAP | 0.088 | 0.035 | 1.092 (1.019-1.169) | 6.283 | **0.012** |
| mPAP | 0.045 | 0.019 | 1.046 (1.008-1.086) | 5.710 | **0.017** |
| CI | -0.529 | 0.296 | 0.589 (0.330-1.053) | 3.191 | **0.074** |
| PVR | 0.151 | 0.022 | 1.016 (0.974-1.061) | 0.568 | 0.451 |
| PAWP | 0.092 | 0.054 | 1.096 (0.986-1.219) | 2.875 | **0.090** |
| CA 125 (category^$^) | 1.684 | 0.411 | 5.389 (2.408-12.059) | 16.798 | **<0.001** |

BPA, balloon pulmonary angioplasty; CA 125, Carbohydrate antigen 125; CI, Cardiac index; CTEPH, Chronic thromboembolic pulmonary hypertension; HR, Hazard ratio; LA, Left atrium dimension; LVED, Left ventricular end-diastolic diameter; LVEF, Left ventricular ejection fraction; ln, logarithmically transformed; mPAP, Mean pulmonary arterial pressure; mRAP, Mean right atrial pressure; NT-proBNP, N-terminal pro-brain natriuretic peptide; PAWP, Pulmonary arterial wedge pressure; PEA, Pulmonary endarterectomy; PVR, Pulmonary vascular resistance; RVED, Right ventricular end-diastolic diameter; 6MWD, 6-min walk distance; sPAP, Systolic pulmonary arterial pressure; S_V_O_2_, Mixed venous oxygen saturation; WHO-FC, World Health Organization functional class. ^$^ CA 125 is classified into two groups, namely CA 125 ≤35 U/ml and CA 125 >35 U/ml.

Table S5 Univariate cox analysis of proportional risks for one-year clinical worsening in patients with IPAH.

| Variable | β | standard error | HR (95% CI) | Wald | *P* value |
| --- | --- | --- | --- | --- | --- |
| Age | 0.012 | 0.011 | 1.012 (0.990-1.034) | 1.088 | 0.297 |
| Female gender | 0.223 | 0.370 | 1.250 (0.605-2.580) | 0.364 | 0.546 |
| 6MWD | -0.002 | 0.001 | 0.998 (0.996-1.001) | 1.289 | 0.256 |
| ln (NT-proBNP) | 0.106 | 0.106 | 1.111 (0.903-1.368) | 0.990 | 0.320 |
| WHO-FC | 0.131 | 0.289 | 1.140 (0.647-2.007) | 0.205 | 0.651 |
| Smoking | -0.140 | 0.522 | 0.870(0.312-2.420) | 0.072 | 0.789 |
| Alcohol intake | 0.061 | 0.522 | 1.063(0.382-2.959) | 0.014 | 0.907 |
| Systemic hypertension | 0.080 | 0.387 | 1.083(0.507-2.314) | 0.043 | 0.836 |
| Diabetes mellitus | -0.369 | 1.011 | 0.691 (0.095-5.010) | 0.134 | 0.715 |
| Hyperlipidemia | 0.328 | 0.596 | 1.388 (0.431-4.467) | 0.302 | 0.582 |
| LVEF | 0.002 | 0.023 | 1.002 (0.957-1.048) | 0.005 | 0.945 |
| LA | 0.007 | 0.037 | 1.007 (0.937-1.083) | 0.036 | 0.850 |
| LVED | -0.005 | 0.025 | 0.995 (0.947-1.046) | 0.040 | 0.842 |
| RVED | -0.011 | 0.023 | 0.989 (0.945-1.034) | 0.250 | 0.617 |
| sPAP | 0.000 | 0.006 | 1.000 (0.989-1.011) | 0.001 | 0.981 |
| Pericardial effusion | 0.033 | 0.409 | 1.033 (0.463-2.303) | 0.006 | 0.937 |
| S_V_O_2_ | -0.055 | 0.021 | 0.946 (0.908-0.986) | 6.814 | **0.009** |
| mRAP | 0.088 | 0.031 | 1.092 (1.028-1.160) | 8.179 | **0.004** |
| mPAP | -0.003 | 0.011 | 0.997 (0.997-1.018) | 0.061 | 0.805 |
| CI | -0.028 | 0.144 | 0.973 (0.733-1.291) | 0.037 | 0.848 |
| PVR | 0.001 | 0.027 | 1.001 (0.950-1.055) | 0.002 | 0.961 |
| PAWP | 0.037 | 0.046 | 1.038(0.948-1.136) | 0.642 | 0.423 |
| CA 125 (category^$^) | 0.599 | 0.334 | 1.820 (0.946-3.501) | 3.219 | **0.073** |

IPAH, Idiopathic pulmonary arterial hypertension; CA 125, Carbohydrate antigen 125; CI, Cardiac index; HR, Hazard ratio; LA, Left atrium dimension; LVED, Left ventricular end-diastolic diameter; LVEF, Left ventricular ejection fraction; ln, logarithmically transformed; mPAP, Mean pulmonary arterial pressure; mRAP, Mean right atrial pressure; NT-proBNP, N-terminal pro-brain natriuretic peptide; PAWP, Pulmonary arterial wedge pressure; PVR, Pulmonary vascular resistance; RVED, Right ventricular end-diastolic diameter; 6MWD, 6-min walk distance; sPAP, Systolic pulmonary arterial pressure; S_V_O_2_, Mixed venous oxygen saturation; WHO-FC, World Health Organization functional class. ^$^ CA 125 is classified into two groups, namely CA 125 ≤35 U/ml and CA 125 >35 U/ml.

Table S6 Multivariate cox analysis of proportional risks for one-year clinical worsening in patients with CTEPH.

| Model^#^ | Variable | β | HR (95% CI) | *P* value |
| --- | --- | --- | --- | --- |
| 1 | CA 125 (category^$^) | 1.696 | 5.450(2.408-12.337) | **<0.001** |
|  | Age | 0.003 | 1.003(0.969-1.037) | 0.871 |
| 2 | CA 125 (category^$^) | 1.723 | 5.601(2.222-14.120) | **<0.001** |
|  | BPA or PEA | 0.057 | 1.059(0.416-2.693) | 0.905 |
| 3 | CA 125 (category^$^) | 1.356 | 3.879(1.522-9.884) | **0.005** |
|  | 6MWD | -0.004 | 0.996(0.992-1.001) | 0.132 |
| 4 | CA 125 (category^$^) | 1.531 | 4.621(2.011-10.623) | **<0.001** |
|  | Hyperlipidemia | -0.778 | 0.459(0.103-2.053) | 0.309 |
| 5 | CA 125 (category^$^) | 1.640 | 5.155(2.300-11.551) | **<0.001** |
|  | LVED | -0.051 | 0.951(0.892-1.013) | 0.117 |
| 6 | CA 125 (category^$^) | 1.622 | 5.066(1.880-13.646) | **0.001** |
|  | RVED | 0.006 | 1.006(0.948-1.068) | 0.833 |
| 7 | CA 125 (category^$^) | 1.706 | 5.508(2.427-12.504) | **<0.001** |
|  | sPAP | 0.026 | 1.026(1.009-1.043) | **0.002** |
| 8 | CA 125 (category^$^) | 1.513 | 4.542(1.898-10.867) | **0.001** |
|  | Pericardial effusion | 0.606 | 1.834(0.674-4.986) | 0.235 |
| 9 | CA 125 (category^$^) | 1.513 | 4.542(1.898-10.867) | **0.001** |
|  | S_v_O_2_ | 0.606 | 1.834(0.674-4.986) | 0.235 |
| 10 | CA 125 (category^$^) | 1.594 | 4.923(1.843-13.152) | **0.001** |
|  | mRAP | 0.013 | 1.013(0.934-1.099) | 0.748 |
| 11 | CA 125 (category^$^) | 1.500 | 4.481(1.935-10.378) | **<0.001** |
|  | mPAP | 0.029 | 1.029(0.991-1.069) | 0.140 |
| 12 | CA 125 (category^$^) | 1.561 | 4.762(2.077-10.920) | **<0.001** |
|  | CI | -0.332 | 0.717(0.405-1.270) | 0.255 |
| 13 | CA 125 (category^$^) | 1.608 | 4.992(2.108-11.822) | **<0.001** |
|  | PAWP | 0.028 | 1.028(0.922-1.146) | 0.617 |

BPA, balloon pulmonary angioplasty; CA 125, Carbohydrate antigen 125; CI, Cardiac index; CTEPH, Chronic thromboembolic pulmonary hypertension; HR, Hazard ratio; LVED, Left ventricular end-diastolic diameter; mPAP, Mean pulmonary arterial pressure; mRAP, Mean right atrial pressure; PAWP, Pulmonary arterial wedge pressure; PEA, Pulmonary endarterectomy; PVR, Pulmonary vascular resistance; RVED, Right ventricular end-diastolic diameter; 6MWD, 6-min walk distance; sPAP, Systolic pulmonary arterial pressure; S_V_O_2_, Mixed venous oxygen saturation. ^#^Given 25 patients with CTEPH experienced clinical worsening, we put 2 independent variables in each Cox model. ^$^ CA 125 is classified into two groups, namely CA 125 ≤35 U/ml and CA 125 >35 U/ml.

Table S7 Multivariate cox analysis of proportional risks for one-year clinical worsening in patients with IPAH.

| Model^#^ | Variable | β | HR (95% CI) | *P* value |
| --- | --- | --- | --- | --- |
| 1 | CA 125 (category^$^) | 0.671 | 1.956(0.959-3.993) | **0.065** |
|  | S_v_O_2_ | -0.066 | 0.936(0.889-0.986) | **0.012** |
|  | 6MWD | 0.000 | 1.000(0.997-1.004) | 0.876 |
| 2 | CA 125 (category^$^) | 0.676 | 1.965(0.960-4.025) | **0.065** |
|  | S_v_O_2_ | -0.065 | 0.937(0.890-0.987) | **0.015** |
|  | 6MWD | 0.001 | 1.001(0.997-1.004) | 0.764 |
|  | Age | 0.013 | 1.013(0.989-1.037) | 0.285 |
| 3 | CA 125 (category^$^) | 0.681 | 1.976(0.967-4.037) | **0.062** |
|  | S_v_O_2_ | -0.070 | 9.32(0.883-0.983) | **0.010** |
|  | 6MWD | 0.000 | 1.000(0.997-1.003) | 0.995 |
|  | ln (NT-proBNP) | -0.064 | 0.938(0.746-1.179) | 0.582 |
| 4 | CA 125 (category^$^) | 0.727 | 2.069(0.993-4.311) | **0.052** |
|  | S_v_O_2_ | -0.068 | 0.934(0.886-0.984) | **0.011** |
|  | 6MWD | 0.000 | 1.000(0.997-1.004) | 0.922 |
|  | Pericardial effusion | -0.275 | 0.759(0.307-1.878) | 0.551 |

IPAH, Idiopathic pulmonary arterial hypertension; ln, Logarithmically transformed; NT-proBNP, N-terminal pro-brain natriuretic peptide; 6MWD, 6-min walk distance; S_V_O_2_, Mixed venous oxygen saturation. ^#^Given 48 patients with IPAH experienced clinical worsening, we put a maximum of 4 independent variables in each Cox model. ^$^ CA 125 is classified into two groups, namely CA 125 ≤35 U/ml and CA 125 >35 U/ml.
