# Supplementary figures and images for: Carbohydrate Antigen 125 Is a Biomarker of the Severity and Prognosis of Pulmonary Hypertension
Source: Front Cardiovasc Med. 2021 Jul 20;8:699904. doi: 10.3389/fcvm.2021.699904 (PMC8330972; doi:10.3389/fcvm.2021.699904)

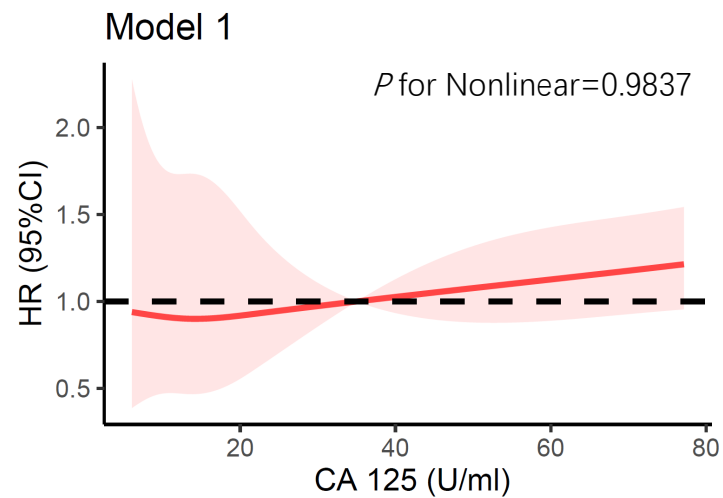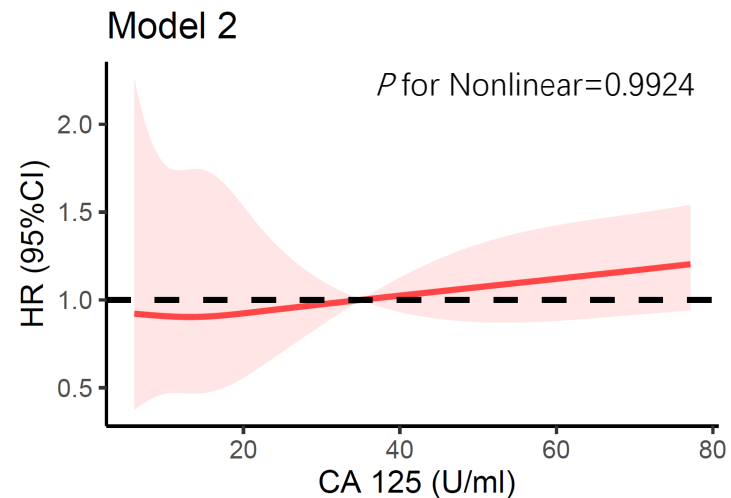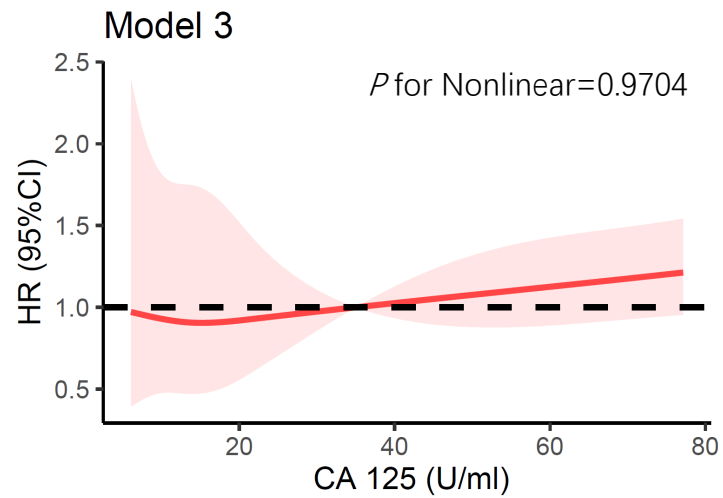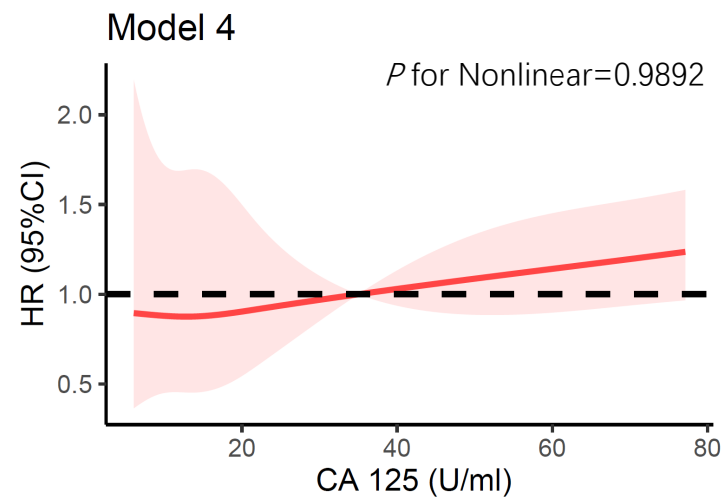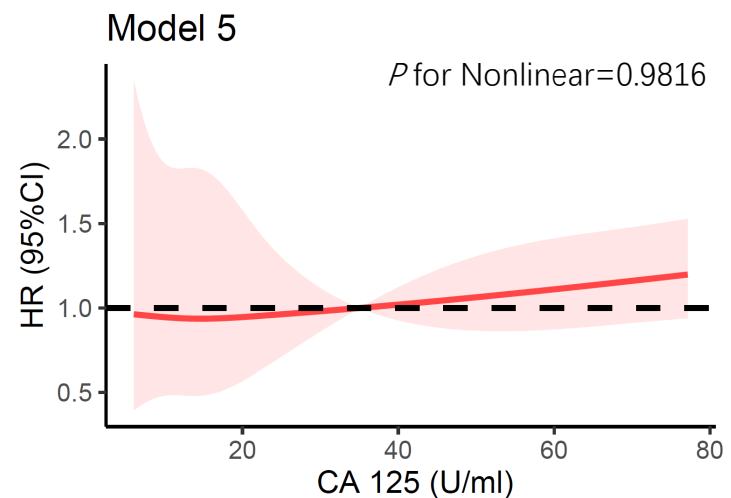

Supplement: Supplementary file 2 [file Image_1.pdf]
